# Accuracy and actionability of mold PCR on bronchoalveolar lavage fluid for diagnosis of invasive mold disease

**DOI:** 10.1128/jcm.01145-25

**Published:** 2025-11-19

**Authors:** Koray K. Demir, Gavin Anderson, Indre Budvytiene, Niaz Banaei

**Affiliations:** 1 Division of Infectious Diseases and Geographic Medicine, Department of Medicine, Stanford University School of Medicine 196261, Stanford, California, USA; 2 Department of Pathology, Stanford University School of Medicine 158566, Stanford, California, USA; 3 Clinical Microbiology Laboratory, Stanford Health Care 474436 , Palo Alto, California, USA; University of Utah, Salt Lake City, Utah, USA

**Keywords:** invasive fungal disease, immunocompromised hosts, mold PCR

## Abstract

Bronchoalveolar lavage fluid (BAL) is commonly obtained for diagnosing invasive mold disease (IMD) in immunocompromised patients. This study investigated the accuracy, turnaround time, and clinical actionability of mold polymerase chain reaction (PCR) on BAL. All BAL tested with mold PCR and fungal culture during the study period were included in this study. The mold PCR detected *Aspergillus* species, Mucorales agents, *Fusarium* species, and *Scedosporium* spp./*Lomentospora prolificans*. Cases were classified per the EORTC/MSGERC definitions. The sensitivity, specificity, turnaround time, and actionability of the mold PCR were measured. Out of 1,958 BAL samples tested, mold PCR was positive in 160 (8.2%) and culture in 75 (3.8%). Mold PCR was most commonly positive for *Aspergillus* species (77.5%), followed by *Scedosporium* spp./*L. prolificans* (20.0%), Mucorales agents (8.3%), and *Fusarium* species (1.2%). The sensitivity and specificity of the mold PCR using culture as the reference standard were 92.0% (69/75, 95% CI 83.4–97.1) and 95.2% (1794/1883, 95% CI 94.2–96.2), respectively. In 32 proven IMD cases, the sensitivity of mold PCR panel and culture was 90.6% (29/32, 95% CI 74.9–98.1) and 56.3% (18/32, 95% CI 37.7–73.7) (*P* < 0.001), respectively. The median time to positivity for mold PCR and culture was 1 day (interquartile range [IQR] 1–2) and 3 days (IQR 2–4) (*P* < 0.001), respectively. Among positive mold PCR results that provided a new finding (*n* = 91), 65.9% were clinically actionable. Mold PCR performed on BAL was more sensitive and rapid compared with fungal culture, and positive results were highly actionable.

**IMPORTANCE:**

Invasive mold disease (IMD) in immunocompromised patients is associated with high morbidity and mortality. Bronchoalveolar lavage fluid (BAL) is commonly obtained for diagnosing pulmonary IMD, but rapid and accurate diagnosis of IMD is challenging with conventional diagnostics. The aim of this study was to investigate the accuracy, turnaround time, and clinical actionability of a BAL mold PCR assay detecting the five most common genera causing IMD. The BAL mold PCR was highly sensitive and specific compared to BAL fungal culture used as the reference method. In patients with proven pulmonary IMD, the BAL mold PCR was highly sensitive and significantly more sensitive compared with fungal culture. The BAL mold PCR was significantly more rapid than fungal culture, and the results were highly actionable. Overall, findings indicate the BAL mold PCR is highly accurate, rapid, and actionable for diagnosis of pulmonary IMD.

## INTRODUCTION

Invasive mold disease (IMD) is associated with high morbidity and mortality in immunocompromised patients, but rapid and accurate diagnosis of IMD is challenging (1). Bronchoalveolar lavage is commonly performed in patients with suspected IMD to diagnose fungal infection. Positive fungal culture results from bronchoalveolar lavage fluid (BAL) can support the diagnosis of probable IMD per the European Organization for Research and Treatment of Cancer and the Mycoses Study Group Education and Research Consortium (EORTC/MSGERC) definitions, but fungal culture has a low sensitivity and can take several days (2, 3). Detection of *Aspergillus* galactomannan in BAL also serves as mycological evidence for probable invasive aspergillosis but does not assist with diagnosis of non-*Aspergillus* molds, and false-positive results are frequently encountered (4). Thus, a more accurate and rapid diagnostic modality on BAL is needed for the diagnosis of IMD.

Polymerase chain reaction (PCR) on BAL is commonly used for diagnosis of invasive aspergillosis. *Aspergillus* PCR performed on BAL has been shown to be highly sensitive and specific in patients with proven and probable invasive aspergillosis and is included in the EORTC/MSGERC diagnostic criteria for probable invasive aspergillosis (2, 4). Mucorales PCR on BAL has also been shown to be highly sensitive in patients with proven and probable mucormycosis but is currently not included in the EORTC/MSGERC diagnostic criteria (5). However, the clinical accuracy of *Aspergillus* and Mucorales PCR in BAL can vary depending on the patient population and the assay used for testing (6, 7). Furthermore, the performance of PCR for detection of other common causes of IMD, such as *Fusarium* spp., *Scedosporium* spp., and *Lomentospora prolificans,* has not been evaluated. In 2020, Stanford Health Care began to offer a laboratory-developed mold PCR panel targeting *Aspergillus* spp., Mucorales agents, *Fusarium* spp., and *Scedosporium* spp./*L. prolificans* (8). This assay is routinely ordered on BAL in addition to fungal culture in patients with suspected IMD. The goal of this study was to assess the sensitivity and specificity, turnaround time, and clinical actionability of BAL mold PCR to assess its added value when ordered in addition to culture.

## MATERIALS AND METHODS

### Study design

A retrospective study was conducted between October 2020 and May 2024 at Stanford Health Care in adult patients with BAL fluid tested with both fungal culture and mold PCR. If a patient had multiple BAL samples in the study interval, each BAL was included separately. The mold PCR was ordered by providers and detects *Aspergillus* spp. (*A. fumigatus, A. flavus, A. niger, A. terreus, A. ustus,* and *A. nidulans*), Mucorales agents (*Rhizopus, Rhizomucor,* and *Mucor* spp.), *Fusarium* spp., and *Scedosporium* spp. (including *L. prolificans*, formerly known as *S. prolificans*) all in one assay. The study interval started when mold PCR became orderable on BAL. Initially, the PCRs for *Aspergillus* spp. and Mucorales agents were orderable individually, before becoming combined into a single panel known as mold PCR. The primary endpoint was mold PCR sensitivity and specificity using fungal culture as the reference standard. Secondary endpoints included mold PCR and fungal culture sensitivity in patients with proven IMD, time to positivity of mold PCR compared with fungal culture, and clinical actionability of positive mold PCR results.

### Clinical and laboratory data

An electronic report was used to determine which BAL samples were tested with both mold PCR and fungal culture. Another electronic report was used to identify all patients who had lung tissue with positive mold results by culture, mold PCR, and/or fungal sequencing. For patients with positive BAL mold PCR and/or fungal culture results and patients with proven IMD but negative BAL mold PCR and fungal culture results, medical records were reviewed to collect clinical and laboratory data, including demographic information, type of immunosuppression, underlying diagnosis and radiographic findings, time to positivity of BAL mold PCR and fungal culture, histopathology results, and whether the clinicians acted on the positive BAL mold PCR result. Using this information, the cases were classified as proven, probable, possible, and no IMD based on the EORTC/MSGERC definitions (2). Patients lacking radiographic and/or clinical data were deemed to have unclassified IMD status. Clinical actionability was defined as a management decision based on the positive mold PCR result as described in provider notes stating that the PCR result was used in clinical decision making such as starting, stopping, or modifying antifungal therapy. If the provider waited for more information rather than just the PCR result, such as a preliminary culture result or other tests, the PCR result was considered non-actionable.

### Mold PCR

A laboratory-developed mold PCR test was performed on weekdays in the Stanford Health Care clinical microbiology laboratory using assay parameters as previously described (8). Briefly, 0.2 mL of BAL was extracted using the EZ2 DNA Tissue Kit (Qiagen, Germantown, MD) according to the manufacturer’s instructions. Singleplex PCR reactions for *Aspergillus* spp. (detecting *A. fumigatus*, *A. flavus*, *A. niger*), Mucorales agents (detecting *Rhizopus* spp., *Rhizomucor* spp., and *Mucor* spp.), and *Scedosporium* spp./ *L. prolificans*, and a multiplex reaction for *A. terreus*, *A. ustus*/*A. nidulans*, and *Fusarium* spp. were performed on the Rotor-Gene 6000 real-time cycler (Qiagen, Germantown, MD) using the primers and probes and PCR conditions as previously described (8). A separate singleplex reaction for the human beta-globin gene was performed as an internal control to assess for DNA extraction and PCR inhibition, and a cycle threshold (CT) of ≤40 was required for results to be considered valid. A CT value of ≤45 was interpreted as a positive result for fungal targets. Positive Mucorales PCR results with CT between 40 and 45 were repeated to confirm results, and positive *A. terreus*, *A. ustus*/*A. nidulans*, and *Fusarium* spp. results were confirmed with singleplex PCR.

### Fungal culture

Fungal culture on BAL was performed on potato flake agar and incubated at 30°C for 3 weeks. Cultures were reviewed daily during the first week, excluding the weekend. Preliminary negative results were reported after the first observation. Cultures with a single colony of mold on the periphery of the agar plate and cultures with *Penicillium* species were adjudicated as being contaminated. The first detectable growth of mold prior to species identification was used to calculate time to positivity.

### Statistical analysis

The sensitivity and specificity of BAL mold PCR was calculated using fungal culture as the reference standard. The sensitivity of BAL mold PCR and fungal culture was also measured using proven IMD as the reference standard. Differences in proportions were evaluated using a Chi-square test, with a P-value <0.05 used as the threshold for statistical significance.

## RESULTS

In total, 1958 BAL samples tested by both fungal culture and mold PCR were included in this study. Among them, 1,794 (91.6%) had a negative result by both fungal culture and mold PCR, and 164 samples from 127 unique patients had a positive result by fungal culture and/or mold PCR. Clinical and demographic data for patients with positive fungal culture and/or mold PCR are presented in Table 1. The median age was 63 years (IQR 49–69), 44.8% (57) were female, and 91.3% (116) were immunosuppressed. The most common immunosuppressed state was solid organ transplantation (42.2%), followed by hematologic malignancy (20.7%), solid organ cancer (10.3%), stem cell transplantation (9.5%), rheumatologic disease (8.6%), and other conditions (8.6%). The IMD status of patients at the time of each bronchoscopy based on the EORTC/MSGERC definitions was 29 (17.7%) proven, 62 (37.8%) probable, 30 (18.3%) possible, 41 (25.0%) no IMD, and two unclassified (Table 1). Prior to receiving a PCR or culture result, 44.1% (56/127) of patients were on anti-mold prophylaxis or treatment, of which 58.9% (33/56) were active against the culture and/or PCR-positive fungal agent. They consisted of azoles in 21, liposomal amphotericin B in 10, and both an azole and amphotericin B in 2 patients.

**TABLE 1:** Demographics, clinical characteristics, and laboratory results for patients with positive BAL fungal culture and/or mold PCR

Characteristic	No. (%) of patients
Age, years (median, IQR)	63 (49–69)
Female sex (%)	57 (44.8%)
Immunosuppression	116 (91.3%)
Solid organ transplant	49 (42.2%)
Lung	45
Liver	1
Heart	1
Heart–lung	2
Stem cell transplantation	11 (9.5%)
Autologous	2
Allogeneic	9
Hematologic malignancy	24 (20.7%)
Solid organ malignancy	12 (10.3%)
Rheumatologic disease	10 (8.6%)
Other^*a*^	10 (8.6%)
EORTC classification	
Proven	29
Probable	62
Possible	30
Not IFI	41
Unclassified	2

^*a*^ Other immunosuppressed states included end-stage renal disease on dialysis, diabetes mellitus, and neurologic diseases requiring immunosuppressive treatment.

Fungal culture and mold PCR were both positive in 69 BAL samples. Fungal culture was positive but mold PCR was negative in 6 samples, and fungal culture was negative but mold PCR was positive in 89 samples. A total of 80 molds were identified in fungal culture, and 169 mold targets were detected by mold PCR (Table 2). Five BAL samples grew two molds in fungal culture, and 9 BAL were positive for 2 (*n* = 7) and 3 (*n* = 1) mold targets by PCR. The molds recovered in culture and detected by PCR are listed in Table 2. By both culture and mold PCR, *Aspergillus* spp. was the most common mold detected (76.3% [61/80] and 77.5 [131/169], respectively), followed by *Scedosporium* spp./*L. prolificans* (20.0% [16/80] and 13.0% [22/169], respectively), the *Mucorales* agents (2.5% [2/80] and 8.3% [14/169], respectively) and *Fusarium* spp. (1.3% [1/80] and 1.2% [2/169], respectively).

**TABLE 2:** Mold agents recovered in culture and detected by mold PCR in BAL samples^*a*^

Fungal culture positive	No. (%)	Corresponding target detected by mold PCR (% agreement)	EORTC classification no. (%) of proven, probable, possible, and no IMD, and unclassified
*Aspergillus* spp.	61 (76.3)	57 (93.4)	14 (23.0), 27 (44.3), 5 (8.2), 15 (24.6), 0 (0)
*Aspergillus fumigatus*	37 (46.3)	NA	13 (35.1), 18 (48.6), 0 (0), 6 (16.2), 0 (0)
*Aspergillus niger*	10 (12.5)	NA	0 (0), 3 (30.0), 1 (10.0), 6 (60.0), 0 (0)
*Aspergillus terreus*	9 (11.3)	5 (55.6)	0 (0), 4 (44.4), 3 (33.3), 2 (22.2), 0 (0)
*Aspergillus flavus*	4 (5.0)	NA	1 (25.0), 2 (50.0), 0 (0), 1 (25.0), 0 (0)
*Aspergillus spp*. unspecified	1 (1.3)	1 (100)	0 (0), 0 (0), 1 (100), 0 (0), 0 (0)
*Scedosporium*/*Pseudallescheria boydii* complex	1 (1.3)	1 (100)	0 (0), 1 (100), 0 (0), 0 (0), 0 (0)
*Lomentospora prolificans*	15 (18.8)	14 (93.3)	4 (26.7), 8 (53.3), 0 (0), 3 (20.0), 0 (0)
Mucorales agents	2 (2.5)	1 (50.0)	0 (0), 1 (50.0), 0 (0), 1 (50.0), 0 (0)
*Rhizopus* spp.	1 (1.3)	1 (100)	0 (0), 1 (100), 0 (0), 0 (0), 0 (0)
*Cunninghamella* spp.	1^*b*^ (1.3)	NA	0 (0), 0 (0), 0 (0), 1 (100), 0 (0)
*Fusarium* spp.	1 (1.3)	1 (100)	0 (0), 0 (0), 0 (0), 1 (100), 0 (0)
Total	80	74 (92.5)	18 (22.5), 37 (46.3), 5 (6.3), 20 (25.0), 0 (0)

Mold PCR target positive	No. (%)	Corresponding species detected in culture (% Agreement)	EORTC classification No. (%) of proven, probable, possible, no IMD, and unclassified
*Aspergillus* spp.	131 (77.5)	51 (38.9)	22 (16.8), 49 (37.4), 24 (18.3), 34 (26.0), 2 (1.5)
*Aspergillus* spp. (*A. fumigatus*, *A. flavus*, *A. niger*)	125 (74.0)	46 (36.8)	22 (17.6), 48 (38.4), 22 (17.6), 31 (24.8), 2 (1.6)
*Aspergillus terreus*	6 (3.6)	5 (83.3)	0 (0), 1 (16.7), 2 (33.3), 3 (50.0), 0 (0)
*Scedosporium* spp.*/ Lomentospora prolificans*	22 (13.0)	15 (68.2)	4 (18.2), 10 (45.5), 2 (9.1), 6 (27.3), 0 (0)
Mucorales agents	14 (8.3)	1 (7.1)	4 (28.6), 8 (57.1), 1 (7.1), 1 (7.1), 0 (0)
*Fusarium* spp.	2 (1.2)	1 (50.0)	0 (0), 0 (0), 1 (50.0), 1 (50.0), 0 (0)
Total	169	68 (40.2)	30 (17.8), 67 (39.6), 28 (16.6), 42 (24.9), 2 (0)

^*a*^ No., number; NA, not applicable; spp., species.

^*b*^ Was deemed to be a contaminant.

Using BAL fungal culture as the reference standard, BAL mold PCR had a sensitivity and specificity of 92.0% (69/75, 95% CI 83.4–97.1) and 95.2% (1794/1883, 95% CI 94.2–96.2), respectively. Positive and negative predictive values of mold PCR were 43.7% (69/158, 95% CI 35.8–51.8) and 99.7% (1794/1800, 95% CI 99.3–99.9), respectively. At a genus level, the sensitivity of mold PCR was 93.4% (57/61) for *Aspergillus* spp., 93.8% (15/16) for *Scedosporium* spp.*/L. prolificans*, 50% (1/2) for Mucorales agents, and 100% (1/1) for *Fusarium* spp. (Table 2). The one missed Mucorales agent was identified in culture as *Cunninghamella* spp., which the mold PCR does not target. This *Cunninghamella* isolate was deemed to be a contaminant by the treating team in a patient with biopsy-proven invasive aspergillosis.

Sensitivity of mold PCR was also assessed and compared to the sensitivity of fungal culture in 32 BAL samples from 19 patients with proven IMD (Table S1), including three BAL from three patients during the study period who had had negative BAL culture and mold PCR results but positive lung tissue biopsies for mold (two *Aspergillus* spp. and one Mucorales agent). Sensitivity of BAL mold PCR and fungal culture in proven IMD cases was 90.6% (29/32, 95% CI 74.9–98.1) and 56.3% (18/32, 95% CI 37.7%–73.7) (*P* < 0.001), respectively.

The median time to positivity with fungal culture and mold PCR was 3 days (IQR 2–4) and 1 day (IQR 1–2) (*P* < 0.001), respectively. Among the 91 BAL samples with positive mold PCR results for which mold PCR provided new information, results were considered actionable by the provider in 65.9% (60/91) of cases. When reported in the medical records, the reasons for non-actionability included positive mold PCR representing colonization (*n *= 6), analytical false-positive result (*n *= 6), or non-invasive fungal syndromes such as allergic bronchopulmonary aspergillosis (*n *= 3), and waiting for culture data prior to changing management (*n *= 3).

## DISCUSSION

PCR assays targeting the most common causes of IMD, such as *Aspergillus* spp. and Mucorales agents, are emerging as valuable tools for diagnosis of IMD. In this study, a BAL mold PCR assay detecting the five most common genera causing IMD was shown to be highly sensitive and specific when compared to fungal culture used as the reference standard. Furthermore, mold PCR provided results several days prior to growth in culture, and positive results were shown to be actionable in the majority of cases where mold PCR provided new information. Since mold growth in culture takes even longer to identify, rapid detection of mold in BAL with PCR is crucial in immunocompromised hosts, where IMD can progress rapidly into life-threatening disease and delayed therapy with appropriate antifungals is associated with increased mortality (9, 10). A key finding of the current study was that BAL mold PCR was highly sensitive and significantly more sensitive than BAL fungal culture in proven cases of IMD (90.6% vs. 56.3%), which not only illustrates the superior performance of mold PCR over fungal culture but also shows that a negative mold PCR result in the appropriate clinical context can be used to exclude the diagnosis of IMD. Although endobronchial and transbronchial biopsy is performed in some patients in addition to BAL during bronchoscopy, histopathology has been shown to be nonspecific for identifying mold when hyphae are present (11). Furthermore, mold PCR, which had a median turnaround time of 1 day in this study, always preceded histopathology and other specific microbiological results.

The high accuracy of mold PCR in this study is consistent with prior studies on the accuracy of BAL *Aspergillus* and Mucorales PCR (4, 5). The higher sensitivity of PCR compared with culture has clinical implications for early diagnosis of IMD and improving outcomes in high-risk populations. For example, pulmonary mucormycosis is one of the most feared forms of IMD, causing aggressive and rapidly progressive disease with mortality rates that can exceed 80% when diagnosis is delayed (12). A non-invasive serum biomarker such as galactomannan, which is routinely performed in patients with suspected IMD to diagnose invasive aspergillosis, does not exist for Mucorales agents. In this study, the Mucorales PCR target was positive in 14 cases from 14 different patients, while culture was positive in only one case, which is consistent with a prior study showing low BAL culture positivity rate compared with PCR (13). The positive Mucorales PCR results in the current study were highly significant as four culture-negative cases were found to be proven mucormycosis cases, and two other cases were deemed by clinicians to reflect probable mucormycosis based on clinical progression and repeatedly positive results, including plasma cell-free DNA PCR in one and sputum culture in another. Similar to Mucorales cell-free PCR, the findings from the current study also support incorporation of Mucorales BAL PCR into the EORTC/MSGERC definitions for probable IMD for more accurate classification of mucormycosis (14).

The BAL mold PCR results in this study were highly actionable and were used to make a clinical decision in 65.9% of patients with positive results when PCR provided a new finding. The actionability rate was likely an underestimation of the true actionability rate because the narrative documentation by providers in the medical charts varied in quality. Decisions may have been made based on the mold PCR results without documenting them in the chart. Prospective studies are needed to more accurately measure the actionability of BAL mold PCR.

Although mold PCR was highly specific when using fungal culture as the reference standard, 24.9% of positive PCR results occurred in patients with no IMD per the EORTC/MSGERC definitions (Table 2) (2). This figure was 25.0% in patients with positive fungal culture (Table 2). Thus, false-positive results may occur with both PCR and culture. The exact cause of such results is not understood but is often attributed to colonization of the airway (15) and less frequently attributed to contamination of assay reagents with environmental spores and non-invasive fungal syndromes such as allergic bronchopulmonary aspergillosis (16, 17). Clinicians should consider the pre-test probability of fungal infection and adhere to the principles of diagnostic stewardship when ordering BAL mold PCR to mitigate obtaining false results (18). When mold PCR is ordered but positive results are questionable, the combination of plasma/serum cell-free DNA and BAL mold PCR with attention to the burden of mold based on PCR CT, as well as conventional biomarkers, may help to interpret the significance of results and determine the likelihood of IMD (19, 20).

Although the findings of this study on the accuracy of BAL mold PCR are promising, this study had several limitations. First, this study was performed at a single center, which may limit the generalizability of its findings. Different centers have different patient populations and clinical and laboratory practices. Fungal culture yield is dependent on several variables, including fungal burden, empiric antifungal therapy, media inoculated, and the fungal species. Thus, our findings may not apply to other centers that have different clinical and laboratory practices. Second, the retrospective design of this study makes it vulnerable to selection bias. Patients too ill to undergo bronchoscopy may have been excluded. Furthermore, non-invasive mold plasma cell-free PCR, which is available and routinely performed prior to invasive testing at our center, may have spared severely ill patients from bronchoscopy and BAL testing (19). Exclusion of such patients may have biased the findings of this study. Third, lung transplants were overrepresented among solid organ transplant patients and also among all patients in this study. Given that lung transplant patients are at high risk for IMD and undergo frequent BALs, their overrepresentation may have biased our findings. Fourth, fungal culture is an imperfect reference standard and therefore may have led to underestimation of the specificity and positive predictive value of mold PCR. We shed light on this limitation by measuring mold PCR sensitivity using proven IMD as a reference standard and showed that PCR is highly sensitive and significantly more sensitive than culture in proven IMD cases. Lastly, the genera detected by the mold panel represent the most common causative agents of IMD. Mold agents that are not detected by the mold PCR would have been missed in this study, although fungal culture did not identify any such cases.

In summary, BAL mold PCR was highly sensitive and specific when using fungal culture as the reference standard. Mold PCR was highly sensitive and significantly more sensitive than fungal culture in proven IMD cases. Mold PCR had a significantly shorter turnaround time compared with fungal culture, and positive mold PCR results were highly actionable. Overall, BAL mold PCR serves an important role in the diagnosis of pulmonary IMD.

## SUPPLEMENTAL MATERIAL

Supplemental tables jcm.01145-25-s0001.docx Table S1.

ASM does not own the copyrights to Supplemental Material that may be linked to, or accessed through, an article. The authors have granted ASM a non-exclusive, world-wide license to publish the Supplemental Material files. Please contact the corresponding author directly for reuse.
