# Supplementary material for: Accuracy and actionability of mold PCR on bronchoalveolar lavage fluid for diagnosis of invasive mold disease
Source: J Clin Microbiol. 2025 Nov 19;63(12):e01145-25. doi: 10.1128/jcm.01145-25 (PMC12710319; doi:10.1128/jcm.01145-25)
Supplement: Supplemental tables — Table S1. [file jcm.01145-25-s0001.docx]

**Supplementary Table 1.** Clinical and microbiological data of patients with proven invasive mold disease.

| **Study ID** | **Age/Sex** | **Underlying disease** | **Clinical Syndrome** | **Histopathology and Tissue Culture Results** | **BAL Mold PCR Result** | **BAL Culture Result** | **Treatment** | **Outcome** |
| --- | --- | --- | --- | --- | --- | --- | --- | --- |
| 52 | 62M | Lung transplant | Bronchial anastomotic dehiscence | Hyphae on histopathology of lung tissue; culture grew *Aspergillus flavus* | *Aspergillus* spp. | *Aspergillus flavus* | Posaconazole & Caspofungin | Death |
| 73 | 54F | Heart & lung transplant | Pulmonary nodules | Hyphae on histopathology of lung tissue identified as *Aspergillus* spp. by mold PCR | *Aspergillus* spp. | *Aspergillus fumigatus* | Posaconazole changed to isavuconazole | Survived |
| 67 | 54F | Systemic lupus erythematosus | Pulmonary consolidation | Hyphae on cytology of lung tissue; culture grew *Aspergillus fumigatus* species complex | *Aspergillus* spp. | *Aspergillus fumigatus* | Voriconazole | Survived |
| 151 | 83F | Bronchiectasis, past cavitary MAC | Pulmonary cavity | Hyphae on histopathology of lung tissue; culture grew *Scedosporium*  /*Pseudallescheria boydii* complex | *Scedosporium* spp.*/*  *Lomentospora prolificans* | *Scedosporium*  /*Pseudallescheria boydii* complex | Voriconazole | Survived |
| 58 | 27F | ALL | Pulmonary nodules | Hyphae on histopathology of lung tissue identified as Mucorales agent by mold PCR | *Aspergillus* spp. & Mucorales | Negative | Amphotericin B | Survived |
| 36 | 64F | Lung transplant | Pulmonary nodules | Hyphae on histopathology of lung tissue identified as *Aspergillus* spp. by mold PCR | *Aspergillus* spp. | *Cunninghamella* spp.^1^ | Amphotericin B | Death |
| 21 | 55F | Lung transplant | Abnormal explanted lung | Hyphae on histopathology of lung tissue identified as *Scedosporium* spp./*Lomentospora prolificans* by mold PCR | *Scedosporium* spp./  *Lomentospora prolificans* | *Scedosporium*  /*Pseudallescheria boydii* complex | Voriconazole | Survived |
| 127 | 49M | AML | Pulmonary cavity | Hyphae on histopathology of lung tissue identified as Mucorales agent by mold PCR | Mucorales | Negative | Amphotericin B | Survived |
| 37 | 86F | B-cell lymphoma | Pulmonary cavities | Hyphae on histopathology of lung tissue identified as *Aspergillus* spp. by mold PCR | *Aspergillus* spp | Negative | Voriconazole | Survived |
| 159 | 71F | Multiple myeloma | Pulmonary nodules | Hyphae on histopathology of lung tissue; culture grew *Aspergillus fumigatus* species complex | *Aspergillus* spp. | Negative | Posaconazole | Died |
| 13 | 66M | ALL | Pulmonary nodules | Hyphae on histopathology of lung tissue identified as *Aspergillus* spp. by mold PCR | *Aspergillus* spp. | Negative | Isavuconazole | Survived |
| 42 | 15M | ALL | Pulmonary nodules | Hyphae on histopathology of lung tissue identified as *Aspergillus* spp*.* by mold PCR | *Aspergillus* spp. | Negative | Unknown | Unknown |
| 60 | 68M | Diabetes | Pulmonary cavities | Hyphae on histopathology of lung tissue; culture grew *Aspergillus fumigatus* species complex | *Aspergillus* spp. | Negative | Isavuconazole | Survived |
| 155 | 65F | AML | Pulmonary cavities | Hyphae on histopathology of lung identified as Mucorales agent by mold PCR | Mucorales | Negative | Amphotericin B | Death |
| 128 | 67F | DLBCL status post CAR-T cell | Pulmonary infiltrate | Necrotizing granulomas and hyphae on histopathology of lung tissue; culture grew *Aspergillus fumigatus* species complex | *Aspergillus* spp. | *Aspergillus fumigatus* | Unknown | Survived |
| 152 | 25M | ALL | Deep soft tissue abscess | Necrotizing pneumonia on lung histopathology with hyphae identified as Mucorales agent by mold PCR | Mucorales | Negative | Amphotericin B | Death |
| 162 | 51F | Lung transplant | Ulcerated tracheal lesion | Hyphae on histopathology of tracheal tissue identified as *Aspergillus* spp. with mold PCR | Negative | Negative | Posaconazole and inhaled amphotericin B | Died |
| 163 | 58M | Lung transplant | Ulcerated tracheal lesion | Hyphae on histopathology of tracheal tissue identified as *Aspergillus ustus*/*nidulans* with mold PCR | Negative | Negative | Posaconazole | Survived |
| 164 | 73M | MDS and allogeneic HSCT | Pulmonary mass | Hyphae on histopathology of lung tissue identified as Mucorales agent by mold PCR | Negative | Negative | Amphotericin B | Died |

ALL, acute lymphoblastic leukemia; AML, acute myeloid leukemia; DLBCL, diffuse large B-cell lymphoma; CAR T-cell, chimeric antigen receptor t-cell therapy; DM2, diabetes mellitus type 2; MDS, myelodysplastic syndrome; HSCT, haematopoietic stem cell transplantation; MAC, *Mycobacterium avium* complex

^1^This *Cunninghamella* isolate was deemed to be a contaminant by the treating team in a patient with biopsy-proven invasive aspergillosis.
